# Enhanced Z-LDA for Small Sample Size Training in Brain-Computer Interface Systems

**DOI:** 10.1155/2015/680769

**Published:** 2015-10-13

**Authors:** Dongrui Gao, Rui Zhang, Tiejun Liu, Fali Li, Teng Ma, Xulin Lv, Peiyang Li, Dezhong Yao, Peng Xu

**Affiliations:** ^1^Key Laboratory for Neuro-Information of Ministry of Education, School of Life Science and Technology, University of Electronic Science and Technology of China, Chengdu 611731, China; ^2^Center for Information in Bio-Medicine, University of Electronic Science and Technology of China, Chengdu 611731, China

## Abstract

*Background*. Usually the training set of online brain-computer interface (BCI) experiment is small. For the small training set, it lacks enough information to deeply train the classifier, resulting in the poor classification performance during online testing. *Methods*. In this paper, on the basis of Z-LDA, we further calculate the classification probability of Z-LDA and then use it to select the reliable samples from the testing set to enlarge the training set, aiming to mine the additional information from testing set to adjust the biased classification boundary obtained from the small training set. The proposed approach is an extension of previous Z-LDA and is named enhanced Z-LDA (EZ-LDA). *Results*. We evaluated the classification performance of LDA, Z-LDA, and EZ-LDA on simulation and real BCI datasets with different sizes of training samples, and classification results showed EZ-LDA achieved the best classification performance. *Conclusions*. EZ-LDA is promising to deal with the small sample size training problem usually existing in online BCI system.

## 1. Introduction

Brain-computer interface (BCI) could translate brain intention into computer commands, and it has been widely used for cursor control [1], word spelling [2], neurological rehabilitation [3], and so forth. Generally, BCI system consists of stimulus presentation, signal acquisition, feature extraction, and translation modules [4]; among them feature extraction and translation algorithms play important roles for the final BCI performance. Three factors, including heteroscedastic class distribution, small sample size training, and nonstationary physiological signals, should be taken into consideration when selecting the translation algorithms for BCI system. Regarding the translation module, linear discriminant analysis (LDA) is one of the most popular classification algorithms for BCI application due to its simplicity, and it has been widely used in motor imagery-based BCI [5], P300 speller [6], and motion-onset visual evoked potential-based BCI (mVEP-BCI) [7]. Besides, the use of linear discriminant analysis (LDA) for functional near-infrared spectroscopy- (fNIRS-) based BCIs is worth mentioning. LDA has been shown to work effectively for the binary [8] and multiclass [9] classifications of motor imagery signals for the development of fNIRS-based BCIs.

However, LDA is established on the homoscedastic class distribution assumption, which is usually not held for practical BCI application. In order to handle this problem, we proposed an improved method named *z*-score LDA (Z-LDA) [10]. Z-LDA defines the decision boundary through *z*-score utilizing both mean and standard deviation information of the projected data, and evaluation results showed better classification performance could be obtained under the heteroscedastic distribution situation. But Z-LDA does not take into account the small sample size training problem that usually existed in actual online BCI system [11]. When the number of the training samples is small, the estimated classifier tends to be overfitted, resulting in the poor generalization during online testing. Various approaches have been proposed to address this issue [12]. Li et al. designed a self-training semisupervised SVM algorithm to train the classifier with small training data [11]. Xu et al. proposed a strategy which enlarges training set by adding test samples with high probability to improve the classification performance of Bayesian LDA under small sample training situation [13, 14]. The strategy hypothesizes that unlabeled samples with high probability provide valuable information for refining the classification boundary.

In essence, Z-LDA defines the confidence of samples in terms of its position in the estimated distribution, which could be used to update the classifier for the online BCI system. In the current study, we will extend Z-LDA to deal with the small size training problem.

## 2. Materials and Methods

### 2.1. Probability Output of Z-LDA

In LDA [15], the weight sum *y*(*X*) of the unlabeled sample *X* is calculated based on the project vector *W* which is estimated from the training set, and the corresponding prediction label is then determined by the shortest distance between *y*(*X*) and the labels of each class. For Z-LDA [10], we assume that *y*(*X*) of samples in each class follow normal distribution and normalize it through *z*-score as

(1) $$\begin{array}{c} z_{k}=\frac{y\left(X\right)-\mu_{k}}{\sigma_{k}}, \end{array}$$

where *μ*_*k*_, *σ*_*k*_ are the corresponding mean and standard deviation of the weight sum *y*(*X*) for training set *C*_*k*_. Thus, *z*_*k*_ follows standard normal distribution; Z-LDA make the prediction based on the distance between *z*_*k*_ and mean of the standard normal distribution (i.e., 0). Suppose *z* ^*∗*^ is the closest one near to 0; then the unlabeled sample will be classified to training set *C* ^*∗*^. In the binary classification, the decision boundary of Z-LDA is defined as [10]

(2) $$\begin{array}{c} c^{*}=\frac{\left(\sigma_{1}\mu_{2}+\sigma_{2}\mu_{1}\right)}{\left(\sigma_{1}+\sigma_{2}\right)}. \end{array}$$

Generally, the cumulative distribution function of the standard normal distribution is denoted as

(3) Φx=PX<x=12π∫−∞xe−t2/2 dt,−∞<x<+∞.

**Figure 1:**
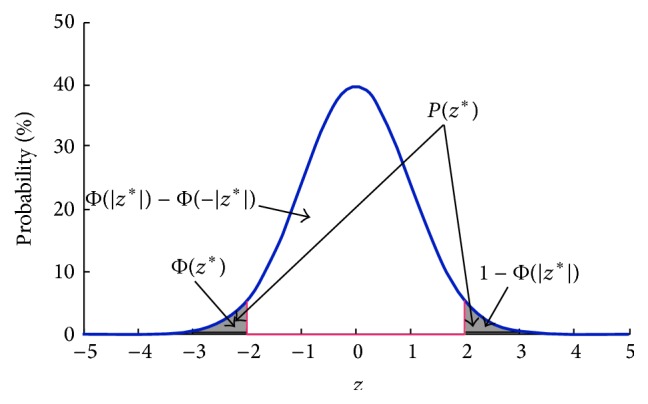
The prediction probability of EZ-LDA and the cumulative probability.

For the transformed *z*-score *z* ^*∗*^ of Z-LDA, the area represents the cumulative probability Φ(*z* ^*∗*^) that is shown in Figure 1. Based on this, we define the prediction probability of Z-LDA as

(4) $$\begin{array}{c} P\left(\;z^{*}\;\right)=1-\left(\;\Phi \left(\;\left|\;z^{*}\;\right|\;\right)-\Phi \left(\;-\left|\;z^{*}\;\right|\;\right)\;\right). \end{array}$$

The area which represents *P*(*z* ^*∗*^) is also marked on Figure 1. It is easy to know that *P*(*z* ^*∗*^) decreases with the increased distance between *z* ^*∗*^ and the mean of the standard normal distribution, and the range of *P*(*z* ^*∗*^) is [0,1]. Obviously, the larger *P*(*z* ^*∗*^) denotes the higher confidence that the sample belongs to class *C* ^*∗*^; thus the above definition is reasonable.

### 2.2. Training Set Enlarging Strategy

**Figure 2:**
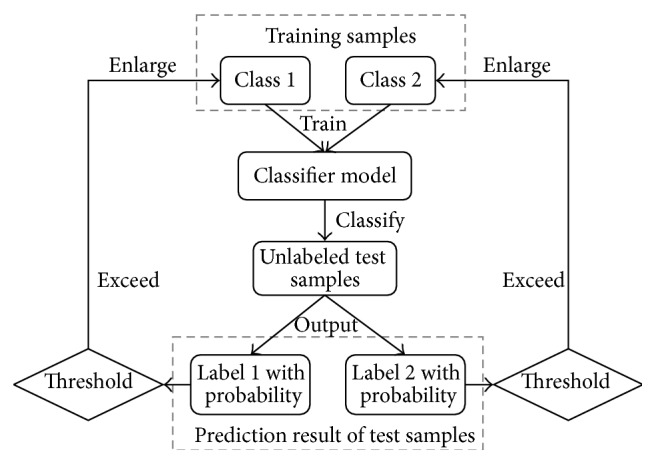
The flow chart of the training set enlarging strategy.

A small training set may not provide enough information for estimating the distribution parameters of the samples; thus the biased classification boundary could be obtained when training the classifier. In this case, the classification accuracy will be decreased during online test. We propose to add a kind of training set enlarging strategy to alleviate the small sample size training effect in this work, and the detailed procedures are illustrated in Figure 2. After classifier model is estimated based on the small training set and the prediction results of unlabeled test samples are obtained, the strategy assumes that unlabeled test samples with high classification probability represent correct prediction, and these correctly predicted samples could be screened out and then used to enlarge the training set. Thus, more accurate sample distribution estimation and the improved classifier with the refined classification boundary would be obtained [13]. In this strategy, classification probability information exported from Z-LDA classifier is regarded as a confident evaluation criterion to select the high probability test samples, which are then labeled according to the prediction results of Z-LDA. Next we need to set a threshold; the predicted label of a test sample is believed to be correct if the corresponding classification probability is higher than the threshold. Finally this test sample could be considered as a training sample because its label has been correctly predicted, and it could be added to the training set for classifier calibration. The above procedures could be repeated several times; thus more samples could be selected, the training set could be enlarged, and the more accurate classification boundary could be found. The above training set enlarging strategy incorporated with Z-LDA is named as enhanced Z-LDA (EZ-LDA) in current study.

### 2.3. Simulations

We constructed a simulation dataset in order to quantitatively investigate the classification performance of EZ-LDA when dealing with the small sample size training problem. The simulation was established by using the fundamental two 2-dimensional Gaussian distributions, where the samples in the first class follow a Gaussian distribution with mean (−5, 1) and standard deviation (1, 1), and the samples in the second class follow a Gaussian distribution with mean (5, −1) and standard deviation (5, 5). 5% outlier samples were added into both the training and testing sets, where the outliers follow a Gaussian distribution with mean (25, −15) and standard deviation (1, 1). There were 50 samples in the training set with 25 samples in each class, and the testing set consisted of 200 samples with 100 samples in each class.

During classifier training, 20, 30, 40, and 50 samples from the training set were selected, respectively. The prediction results of LDA and Z-LDA were also calculated for comparison. Regarding EZ-LDA, the test set was divided into 20 parts, with 10 samples in each part. Then we used the classifier estimated from original training set to predict the labels of the first part (10 samples) of the test set and obtained the predicted label and classification probability of each sample. Next we set a probability threshold; the predicted label of a test sample is believed to be correct if the corresponding classification probability is higher than the threshold. Finally this kind of test sample could be considered as a training sample because its label has been correctly predicted, and it could be added to the training set. Once the first part (10) samples have been processed, we retrain the classifier based on the extended training set; then using the updated classifier to process the next 10 test samples, the repetitions will be stopped until all the test samples are predicted. The probability thresholds ranged from 10% to 90% with a step 10% being considered. The above procedures were repeated 100 times in order to reduce the random effect, and all the samples of the training and testing set were generated at the beginning of each iteration. Finally, the average classification accuracies were obtained for the three classifiers, respectively.

### 2.4. Real BCI Dataset

#### 2.4.1. Dataset Description

The evaluation dataset comes from motion-onset visual evoked potential-based BCI (mVEP-BCI) experiment. mVEP could be evoked by brief motion of object, and it is time locked to the onset of the motion. We can achieve the brief motion stimuli by screen virtual button; thus BCI system based on mVEP could be developed [7, 16].

Eight subjects (2 females, aged 23.3 ± 1.3 years) participated in the current study. They were with normal or corrected to normal vision. The experimental protocol was approved by the Institution Research Ethics Board of the University of Electronic Science and Technology of China. All participants were asked to read and sign an informed consent form before participating in the study. After the experiment, all participants received monetary compensation for their time and effort.

The experimental paradigm is similar as we described in [17]; six virtual buttons were presented on the 14-inch LCD screen, and, in each virtual button, a red vertical line appeared in the right side of the button and moved leftward until it reached the left side of the button, which generated a brief motion-onset stimulus. The entire move took 140 ms, with a 60 ms interval between the consecutive two moves. The motion-onset stimulus in each of the six buttons appeared in a random order, and a trial was defined as a complete series of motion-onset stimulus of all six virtual buttons successively. The interval between two trials was 300 ms; thus each trial lasted 1.5 s. Five trials comprised a block, which costs 7.5 s. The subject needs to focus on the button which is indicated in the center of the graphical user interface, and the instructed number randomly appeared. To increase their attention, the subject was further asked to count in silence the times of moving stimulus appearing in the target button. A total of 144 blocks, including 720 trials, were collected for each subject in four equal separate sessions, with a 2-minute rest period between sessions.

Ten Ag/AgCl electrodes (CP1, CP2, CP3, CP4, P3, P4, Pz, O3, O4, and Oz) from extended 10–20 system were placed for EEG recordings by using a Symtop amplifier (Symtop Instrument, Beijing, China). All electrode impedance was kept below 5 kΩ, and AFz electrode was adopted as reference. The EEG signals were sampled at 1000 Hz and band-pass filtered between 0.5 and 45 Hz.

#### 2.4.2. Preprocessing and Feature Extraction

Since the scalp recorded EEG signals are usually contaminated with noise, those trials with absolute amplitude above 50 *μ*v threshold were removed from the following analysis. The remaining EEG data were band-pass filtered between 0.5 Hz and 10 Hz, because the mVEP is usually distributed in the low frequency band [18]. Then the EEG epochs of 5 trials in each block were averaged by stimulus. Similar to P300-based study, the instructed stimulus was defined as target, and the others were defined as nontarget. Two-sample *t*-test was applied between the target and nontarget epochs to find the channels and time windows which exhibit significant difference. Finally, three significant channels were selected for each subject, and the time windows ranged from 140 ms to 350 ms, which varied between subjects. The selected epochs were further down-sampled to 20 Hz, and a 9-dimensional or 12-dimensional feature vector was generated for each stimulus in the block at the end.

#### 2.4.3. Small Sample Size Classification

The aim of the mVEP-BCI was to distinguish the target and nontarget stimuli, that is, recognizing the button which subject paid attention to; thus it was a binary classification problem. The ratio of the nontarget and target number in each trial was 5 : 1, and we selected equal number of target and nontarget samples for initially training in the following analysis in order to balance the two classes. 20, 40, 60, 80, and 100 samples were used to train the classifiers, respectively, and the remaining samples were used for test. Similar to the strategies demonstrated in Simulations, the higher probability test samples were added into the training set to update the classifier for EZ-LDA with the step also being 10 samples, and the threshold was set as 50%, because the highest accuracy was achieved at this threshold on the simulation dataset. Similarly, the classification results of LDA and Z-LDA were also calculated for comparison.

## 3. Results

**Figure 3:**
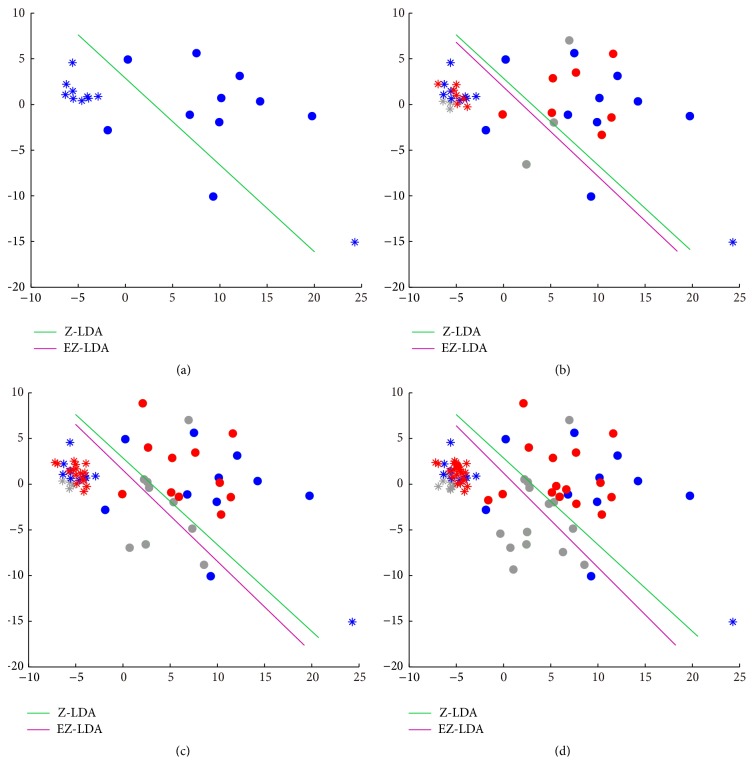
The classification boundary adjustment processes of EZ-LDA. (a) The initial 20 training samples; (b) 20 training samples + 20 test samples; (c) 20 training samples + 40 test samples; (d) 20 training samples + 60 test samples. Star: the samples in the first class; filled circle: the samples in the second class; blue: the samples in the training set; red: the higher probability test samples for extending the training set; grey: the lower probability test samples.

The classification results of the simulation dataset were shown in Table 1, where the accuracies of all the three classifiers increased with the extending of the initial training sample size. EZ-LDA achieved the highest average accuracies under all the sample size conditions, and the accuracies obtained by EZ-LDA under all thresholds were significantly higher than Z-LDA (*p* < 0.05, Mann-Whitney *U* test). When the training sample size was bigger than 20, both Z-LDA and EZ-LDA achieved higher accuracies than LDA. Regarding the various thresholds in EZ-LDA, the best performance was obtained for the threshold 50%. To further reveal the working mechanism of EZ-LDA, an illustration of the training set enlarging strategy was given in Figure 3. The initial training sample size was 20; Z-LDA and EZ-LDA shared the same classification boundary (Figure 3(a)). When the high probability samples in testing set were included in the training set, the corresponding boundary lines of EZ-LDA could be adaptively adjusted (Figures 3(b)–3(d)).

**Figure 4:**
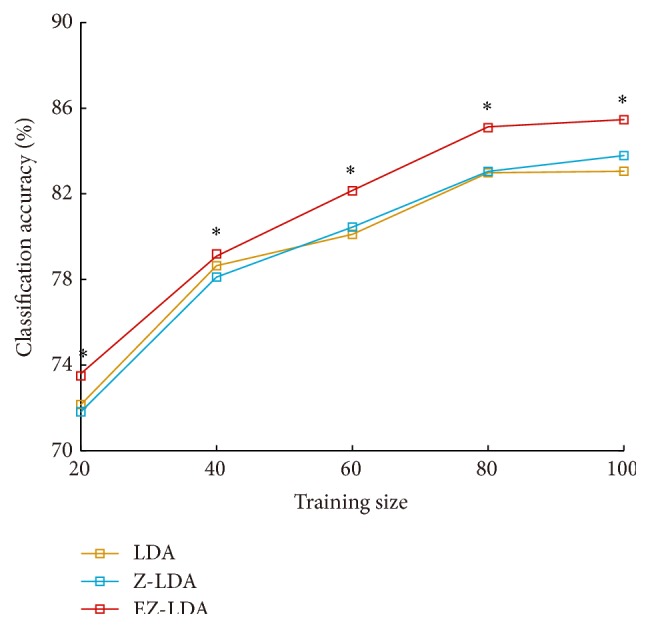
Classification results of LDA, Z-LDA, and EZ-LDA on mVEP-BCI dataset with different training sample sizes. *∗* denotes the classification accuracy of EZ-LDA is significantly higher than that of Z-LDA (Mann-Whitney *U* test, *p* < 0.05).

Figure 4 presented the performances on real mVEP-BCI dataset for LDA, Z-LDA, and EZ-LDA. For all the three kinds of classifiers, the mean accuracies increased when we enlarged the training size, and this is consistent with the simulation result. Moreover, the classification accuracies of EZ-LDA consistently outperformed Z-LDA in all of the five considered training sample sizes (*p* < 0.05, Mann-Whitney *U* test); compared with LDA, the significant improvement could be also observed except the training size with 40 samples.

## 4. Discussion

Translation algorithms in BCI system could translate intent-related features to computer commands, and generally we need to collect a training set at first for training the classifier. But due to the factors such as experiment time limit, electrode conductivity decrease over time, and subject fatigue, usually the training set is not big enough in BCI application. Besides, as for the online BCI system, the subject's mental state may vary largely from the previous mental state during training. Therefore, in order to track subject's mental state change, it is necessary to update the classifier by utilizing the information from those new test samples, which is also useful for resolving the small sample size training problem.

The best way to reduce the bias effect is to include enough samples to train the classifier, but it is unable to achieve in actual BCI experiment. Alternatively, we could consider the unlabeled test samples with high classification probability, as the prediction of them could be trusted. Thus we may enlarge the training set by adding those test samples with high classification probability in testing set to refine the classifier. Results from both simulation and real BCI datasets in current study showed that the enlarging strategy for training set could improve the classification performance of Z-LDA under small sample size situation. The classification boundary definition of Z-LDA is based on the distribution of the training samples; vividly it can be viewed as the intersection of the Gaussian distribution curves [10]. If the training set is too small, we may not get the accurate distribution information of the samples, resulting in the biased classifier boundary. In this case, the classification accuracy of Z-LDA may be decreased as revealed in the results from both simulation and actual BCI datasets. Specifically, in simulation dataset, the mean accuracies of Z-LDA are lower than those of LDA when the training size is 20; similarly in real BCI dataset when 20 or 40 samples are selected for training, the mean accuracies of Z-LDA are lower than LDA. However, EZ-LDA achieved better accuracies than LDA in both simulation and real BCI datasets, but EZ-LDA still needs a number of training samples for initial classifier training; based on the simulation and real BCI datasets in current study, we think the minimum number of training samples is 20 for EZ-LDA to perform well. Adding the test samples with higher classification probability to training set could enlarge the training size; thus more accurate sample distribution information could be estimated for Z-LDA, and the biased classification boundary could be corrected too. As shown in Figure 3, the classification boundaries are the same between EZ-LDA and Z-LDA for the initial training set (Figure 3(a)). Then the boundary is used to predict the labels of the test samples, and those predicted test samples with high classification probability are selected to extend the training set; finally the classification boundary is refined (Figure 3(b)). The above procedures could be repeated again to obtain more accurate classification boundary (Figures 3(c) and 3(d)). Noted that some of the test samples may be wrongly classified during prediction, usually these kinds of samples are far from the distribution center and with lower classification probability, for example, the grey filled circles in Figure 3. Since they may influence the estimated classifier model, we set a threshold to screen them out.

The classification probability of Z-LDA is based on the cumulative distribution function Φ(*x*) of the standard normal distribution. The cumulative probability is equal to 0, 50%, and 100% when *x* are −*∞*, 0, and +*∞*, respectively. Mathematically, Z-LDA measures similarity of how a sample belongs to this distribution by the distance between the distribution center 0 and the sample variable defined in (1). Following (4), the closer to distribution center the sample variable is, the higher probability the sample belongs to the class with this distribution. To expand the training set, we need to set a probability threshold to select the test samples with correctly predicted label. Obviously, more wrongly predicted samples may be added to the training set when a lower threshold was set, whereas when the threshold was set higher, fewer samples could be selected to add in the training set. The biased classification boundary cannot be effectively corrected under the two above conditions, as proved by Table 1 that the relatively poor performance is achieved for EZ-LDA when the threshold is set as 10% or 90%. However, the highest mean accuracy is achieved when the threshold is set as 50%. Therefore, the threshold should be set as a compensation of the number of correctly and wrongly predicted samples in the actual application. In the real BCI dataset, we only considered the 50% threshold for illustration, because the highest accuracies are achieved under this threshold on simulation dataset. As shown in Figure 4, when the enlarging strategy is combined with EZ-LDA, the performance of EZ-LDA has statistical improvement compared with LDA and Z-LDA, because the information in the testing set can be mined to update the classifier.

SVM is an another popular classifier used for BCI application [19, 20]; the advantages of LDA are simplicity and low computational cost, while SVM has better generalization capability. Theoretically, the optimal hyperplane of SVM maximizes the distance between the support vectors (the nearest training points); thus it is highly dependent on the nearest training points which are found from the training samples. However, when there are fewer samples in the training set, the selected nearest training points may not be representative; thus the obtained optimal hyperplane may be biased too. Therefore, SVM cannot solve the small sample size training problem.

## 5. Conclusions

In the current study, we proposed adding the test samples with higher classification probability to the training set for obtaining comprehensive distribution information; thus the biased classification boundary estimated from the small training set could be corrected. The effectiveness of EZ-LDA in handling the small sample size training problem was validated on both simulation and real BCI datasets. EZ-LDA is an extension of Z-LDA, and it is easy to be implemented in real-time BCI systems.

## Figures and Tables

**Table 1:** The classification accuracies of LDA, Z-LDA, and EZ-LDA on simulation dataset.

Training size		20	30	40	50

LDA		78.7	80.4	81.9	83.4

Z-LDA		77.0	81.7	83.0	84.0

EZ-LDA (different thresholds)	10%	81.2^*∗*^	83.6^*∗*^	84.9^*∗*^	85.8^*∗*^
	20%	81.5^*∗*^	83.8^*∗*^	85.1^*∗*^	86.0^*∗*^
	30%	81.6^*∗*^	83.9^*∗*^	85.2^*∗*^	86.3^*∗*^
	40%	81.6^*∗*^	84.0^*∗*^	85.5^*∗*^	86.4^*∗*^
	50%	81.8^*∗*^	84.1^*∗*^	85.5^*∗*^	86.4^*∗*^
	60%	81.4^*∗*^	83.9^*∗*^	85.3^*∗*^	86.2^*∗*^
	70%	81.3^*∗*^	83.6^*∗*^	85.1^*∗*^	86.2^*∗*^
	80%	81.1^*∗*^	83.2^*∗*^	84.8^*∗*^	85.8^*∗*^
	90%	80.3^*∗*^	82.8^*∗*^	84.2^*∗*^	85.2^*∗*^

The second column denotes the threshold used in EZ-LDA.

*∗* denotes the classification accuracy of EZ-LDA is significantly higher than that of Z-LDA (Mann-Whitney *U* test, *p* < 0.05).
